# Computational Modeling of Auxin: A Foundation for Plant Engineering

**DOI:** 10.3389/fpls.2016.01881

**Published:** 2016-12-20

**Authors:** Alejandro Morales-Tapia, Alfredo Cruz-Ramírez

**Affiliations:** Molecular and Developmental Complexity Group, Unidad de Genómica Avanzada, Langebio-CinvestavIrapuato, México

**Keywords:** auxin, computational modeling, development, morphodynamics, plants

## Abstract

Since the development of agriculture, humans have relied on the cultivation of plants to satisfy our increasing demand for food, natural products, and other raw materials. As we understand more about plant development, we can better manipulate plants to fulfill our particular needs. Auxins are a class of simple metabolites that coordinate many developmental activities like growth and the appearance of functional structures in plants. Computational modeling of auxin has proven to be an excellent tool in elucidating many mechanisms that underlie these developmental events. Due to the complexity of these mechanisms, current modeling efforts are concerned only with single phenomena focused on narrow spatial and developmental contexts; but a general model of plant development could be assembled by integrating the insights from all of them. In this perspective, we summarize the current collection of auxin-driven computational models, focusing on how they could come together into a single model for plant development. A model of this nature would allow researchers to test hypotheses *in silico* and yield accurate predictions about the behavior of a plant under a given set of physical and biochemical constraints. It would also provide a solid foundation toward the establishment of plant engineering, a proposed discipline intended to enable the design and production of plants that exhibit an arbitrarily defined set of features.

## Auxin Modeling in An Intracellular Context

A change in auxin concentration induces a change in the transcriptional program of the cell. Auxin response is carried out by the Auxin Response Factor (ARF) family of transcriptional regulators. Due to the variety of ARFs present in plants, the auxin signal can be interpreted differently by cells that belong to different lineages and tissues (Rademacher et al., 2011). Taking all of them into account is necessary to accurately predict a plant’s phenotype, but this would require the simulation of a very complex regulation network. It has been shown, however, that this complexity can be considerably reduced if we identify the dominant players of the network and design a simplified model accordingly (Vernoux et al., 2011).

Auxin is transported from cell to cell through the action of active transporters that are present at the cell’s membrane, such as those belonging to the AUX/LAX and PIN families. This enables many important developmental events (Blilou et al., 2005). These transporter proteins are commonly found polarized toward a preferred region of the membrane, as described in a mathematical model by Kleine-Vehn et al. (2011). The location of these transporters correlates with the amount of auxin present in adjacent cells. Several hypotheses have been proposed to explain this and they have been tested through computational modeling (Wabnik et al., 2011).

A relatively simple model could account for both auxin response and transporter polarization to yield predictions on the distribution of auxin across a larger tissue. Other noteworthy phenomena that could be considered at this scale are: the diffusion of auxin within the cell (Lituiev et al., 2013); auxin transport between the cytoplasm and the nucleus (Herud et al., 2016); the feedback between auxin concentration and the amount of protein regulators like Aux/IAAs (Middleton et al., 2010) and membrane transporters like LAX3 (Mellor et al., 2015); and other mechanisms that are involved with auxin homeostasis (Mellor et al., 2016). All these examples could be integrated into a single model that describes the behavior of auxin from a cellular point of view.

## Auxin Maxima

On a broader scale, as auxin travels across tissues, it tends to accumulate around localized spots which accrue a significantly higher concentration. Auxin maxima, as they are commonly known, usually precede the appearance of organs and other morphological structures, and consequently have been one of the most important traits that computational auxin models aim to recreate.

At the tip of *Arabidopsis thaliana* roots, a maximum of auxin maintains the identity of a group of cells known as the root stem cell niche (RSCN). A model by Grieneisen et al. (2007) reproduces the auxin dynamics in this region and considers auxin transport due to efflux membrane transporters (i.e., PIN proteins). The model represents the root as a 2-dimensional lattice of square cells which have different permeability values on their sides. With a similar setup, Tian et al. (2014) showed that the presence of WOX5, involved in auxin biosynthesis, is necessary for the appearance of this auxin maximum. Another study by Band et al. (2014) makes use of a realistic layout of cells and further extends the model by accounting for the effect of auxin influx transporters (i.e., AUX1/LAX).

It is crucial to consider the properties of auxin transporters when modeling the auxin flow that travels through a group of cells (Mitchison, 2015). Other mechanisms that regulate transporters themselves need to be considered as well. In *A. thaliana*, ICR1 and a family of related proteins control the deposition of PIN proteins on the cell membrane (Hazak et al., 2010). Their activity is dependent on the amount of auxin present within the cell and they are able to alter the resulting auxin distribution pattern of the tissue to which they belong (Hazak et al., 2014).

Regardless of the completeness of a single cellular model of auxin development, auxin maxima are emergent spatial features which cannot be seen if cells are considered as isolated entities. Their location, timing, and other physiological properties are the result of a complex system of cells interacting together and these characteristics are heavily influenced by the geometrical arrangement of neighboring cells and the phenomena associated with auxin transport from cell to cell.

## Patterning and Growth

Auxin coordinates the development of many different structures that a plant needs to function, thrive, and reproduce (Vanneste and Friml, 2009). This is accomplished mainly through mechanisms that involve cell elongation, division, and differentiation; all of which are studied through computational modeling.

The distribution of cells found at the *A. thaliana* root tip can be explained by a model where cell division is controlled by a biological clock that depends on the amount of auxin present (Barrio et al., 2013), as well as specific developmental rules that seek to, among other things, balance out the total strain exerted by the growing tissue (De Vos et al., 2014).

In the *A. thaliana* RSCN, asymmetric cell division of the Cortex and Endodermis Initial (CEI) is controlled by the active form of the SCR/SHR protein complex (Di Laurenzio et al., 1996; Sabatini et al., 2003; Cui et al., 2007). RBR protein binds to, and inactivates, the SCR/SHR complex, unless prevented by CYCD6;1, which is expressed only in the CEI due to the existing auxin maximum at the RSCN. As cells move away from the auxin maximum and are, thus, exposed to a lower auxin concentration, RBR can deactivate SCR/SHR and prevent further asymmetric division of the daughter cells (Cruz-Ramírez et al., 2012).

Also in the root, the PLT family of transcription factors follows a concentration gradient that increases toward the tip and controls the growth of cells at the elongation zone. While the presence of PLT is relatively uniform and symmetric, the auxin gradient present is sensitive to changes in the physical orientation of the root structure. A model by Mähönen et al. (2014) describes how, through the integration of these two signals, the plant can effect different elongation rates on different regions of the root, allowing it to carry out an adequate gravitropic response.

Phyllotaxis is a particular pattern of organization, resembling a spiral, that is exhibited by many biological structures such as leaves, and the characteristic arrangement of seeds in a sunflower. Early studies involving auxin and its relationship with phyllotaxis have been carried out by Jönsson et al. (2006) and Smith et al. (2006). These studies locate auxin transporters within a given cell depending on the auxin concentration present in adjacent cells. This results in the repeated appearance of auxin maxima yielding a pattern that heavily resembles a phyllotactic arrangement. The phyllotactic pattern has also been found to be involved, and modeled, in the organization of floral primordia (van Mourik et al., 2012); and also influencing the development of leaves and their resulting shape (Chitwood et al., 2012).

Many other examples of computational auxin models applied to the study of patterning events exist. Jones et al. (2009) made use of a mathematical model to estimate the amount of auxin present in root hair cells; Bilsborough et al. (2011) showed how the appearance of auxin maxima spots along the edge of leaves gives rise to their resulting serration pattern; Fujita et al. (2011) explored the variety of arrangements arising from the shoot meristem of plants; Mirabet et al. (2012) studied the extent to which the phyllotactic pattern is vulnerable to naturally occurring fluctuations; Péret et al. (2013) proposed a mechanism that explains how lateral roots may emerge from the primary root structure; and Fàbregas et al. (2015) described a model that recreates the position and number of vascular bundles that appear in the developing shoot.

All these models further push the spatial context of auxin simulations into a larger realm that allows the study of the intended physiological function of biological structures. They are also built up from a common set of premises, particularly similar in regard to how they implement auxin transport between cells. The variety of biological functions that had been reproduced using this relatively small set of rules provides a strong argument in favor of a general model of plant development that relies on auxin as a common integrating signal.

## Canalization Models

Plants rely on specialized structures that irrigate tissues with water and nutrients in order to function properly. The organization and function of these vascular tissues is controlled by a patterning event that involves auxin and its transport. When auxin flows through a tissue, the auxin transporters in its composing cells reorient toward the direction of the broader auxin flux. This leads to the establishment of preferential canals where auxin is transported along, a phenomenon known as canalization.

Early models involving canalization were proposed by Feugier et al. (2005), Rolland-Lagan and Prusinkiewicz (2005), Fujita and Mochizuki (2006a,b), Prusinkiewicz et al. (2009), and Wabnik et al. (2010), who all showed that the venation patterns can be produced through stable and self-organizing mechanisms. With different locations for auxin sources and sinks, an extensive variety of vascular arrangements can be generated. Cell division also needs to be carefully controlled in cells that are part of the emerging canal, so as to maintain the continuity of the venation pattern (Lee et al., 2014).

Canalization models are needed to incorporate into exhaustive models of plant development, as they explain how a plant can promote and maintain its functional structures. A study by O’Connor et al. (2014), takes a step in this direction, showing how vascularization and organ initiation are closely related events carried out by the concerted action of distinct PIN proteins. This study also extends the validity of the canonical auxin/PIN mechanism into a broader phylogenetic context, since they used *Brachypodium distachyon* as the model organism.

## Structural Mechanics

Mechanical stress across tissues plays an important role during the life of a plant. This stress can be perceived by cells as it travels through their internal structure and it is known to induce change in the organization of the cell cytoskeleton (Hamant et al., 2008) and alter the polarization of auxin transporters (Heisler et al., 2010). Complementary to that, developmental effects resulting from auxin response alter the shape and other structural properties of a growing plant, hinting at a system where auxin and mechanical forces continuously interact with each other.

Auxin can induce changes in the pH of the apoplastic space, as thoroughly explained by a model from Steinacher et al. (2012). When the pH on the environment of a cell wall drops, its structural strength is diminished, allowing it to change its shape and yield to other forces like turgor pressure. This is a mechanism that allows the plant to control the anisotropic growth of tissues through the presence of varying concentrations of auxin (Sassi et al., 2014).

Mechanical forces are part of another theoretical layer that would greatly expand the scope of computational models of plant development. Unfortunately, accurate measurements on the mechanical properties of plant cells and tissues are still scarce. Nevertheless, recent efforts are producing valuable parameters; for example, Beauzamy et al. (2015) estimated the cell wall’s stiffness and turgor pressure at the *A. thaliana* shoot meristem.

## Pathway Crosstalk

Further extending the influence of auxin throughout plant development, auxin is known to interact with other hormonal pathways. For example, cytokinins (CK) are known to have a particularly close relationship with auxin. The auxin/CK antagonistic interplay has been studied by Muraro et al. (2011), who predict a dynamic which can switch from a bistable equilibrium system to a system that exhibits oscillatory changes, leading to different developmental consequences for the plant. This model was used to recreate the architecture of the *A. thaliana* root by Muraro et al. (2013). The relationship between auxin and CK also is involved with other physiological features like miRNA regulation (Muraro et al., 2014; el-Showk et al., 2015) and the geometrical distribution of the developing cells (De Rybel et al., 2014).

Auxin and brassinosteroids (BRs) work together in the formation of vascular tissue inside the *A. thaliana* shoot. This tissue is directly specified by the presence of regularly spaced auxin maxima regions along the shoot. However, with different levels of BRs, the cells at the inflorescence stem change in number and size, which influences the resulting pattern of auxin maxima and, consequently, the number of vascular bundles that appear (Ibañes et al., 2009). Strigolactone is another hormone that interacts with auxin, as it is known to promote the dissociation of PIN1 from the membrane and, thus, alter the effective rate of auxin transport. This is linked to developmental features like shoot branching in *A. thaliana* (Shinohara et al., 2013).

Sometimes, multiple hormones can interact with auxin to produce a single phenotype. For instance, at the *A. thaliana* root, the observed auxin distribution pattern is known to be affected by a complex regulation network involving CK, ethylene, and the PLS protein (Liu et al., 2010; Moore et al., 2015).

The above are all prime examples of how auxin models could be extended to incorporate other regulation mechanisms that are important for plant development. A computational model intended to reproduce as much as we know about the metabolism of plants, should consider auxin as a starting point, due to the extensive work that has been published and is available to date.

## Tropic Response

Plants can sense external stimuli from their environment and react to it by changing their developmental program. For example, regarding the gravitropic response in the *A. thaliana* root, the signal originates at the root tip, increasing the local auxin concentration in the tissue following the orientation of the gravitropic stimulus. This pulse, then, travels through the root to deliver the signal to the elongation zone, where the appropriate response is carried out (Mähönen et al., 2014). Band et al. (2012) devised a mathematical model to estimate the speed of auxin redistribution in response to changes in the gravity vector. Swarup et al. (2005) showed how the auxin pulse is transported through the epidermis, due to the presence of PIN and AUX/LAX membrane transporters.

A physical deformation exerted over a tissue is enough to trigger a tropic response from the plant, as it is known that roots are more prone to spawn lateral roots in regions that were previously bent. A convincing explanation for this is given in Laskowski et al. (2008), which predicts that modifying the shape of the root at the meristematic zone leads to an increase in auxin toward the outer region of the curve. This causes an increased accumulation of AUX1 transporters in pericycle cells and induces their reprogramming into lateral root founder cells.

Tropic response in computational models provides an environmental layer which greatly influences the development of a plant. Auxin is known to play a fundamental role in the plant’s response to gravity, light, humidity, and other environmental cues (Retzer et al., 2014). It would be reasonable, then, to consider auxin a fundamental signal that enables this environmental layer to interact with the remaining parts in a model of plant development.

## Toward A Single Model of Plant Development

**FIGURE 1:**
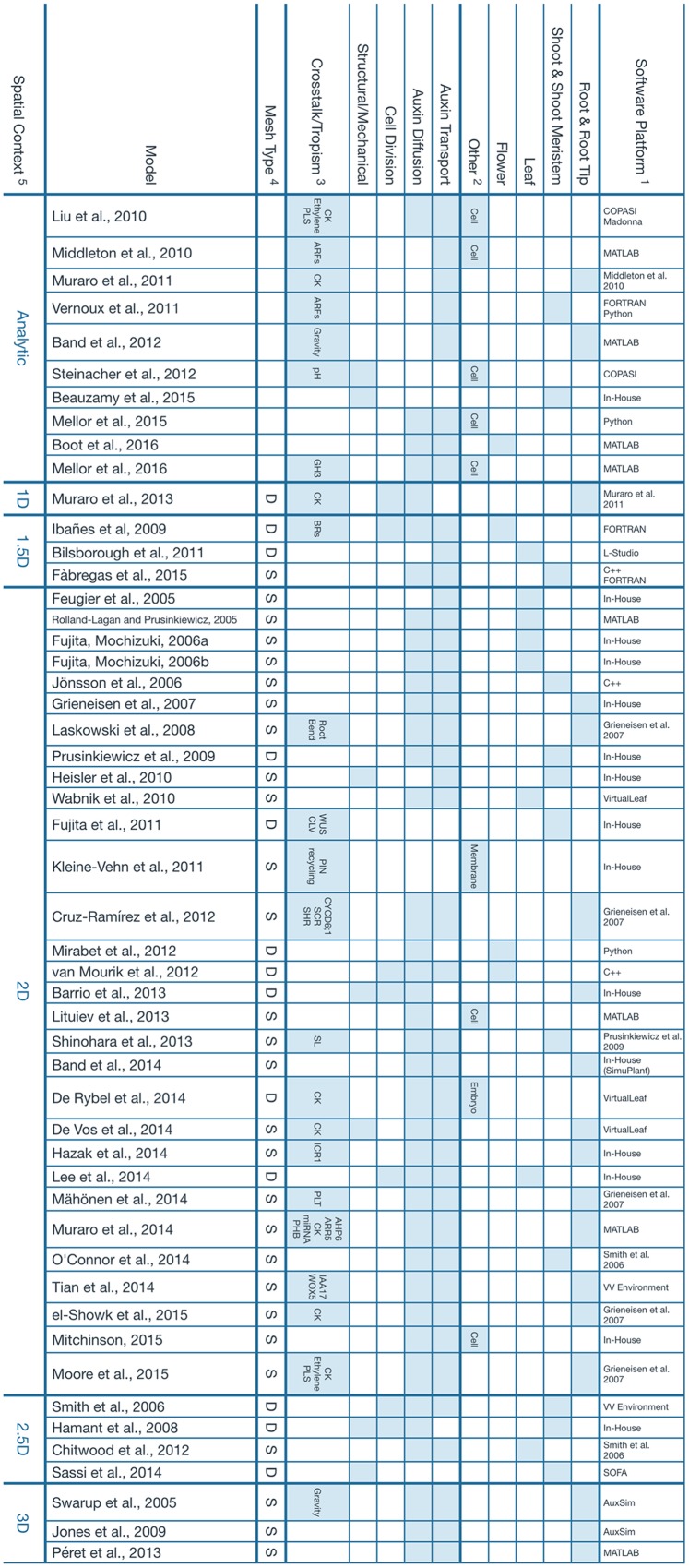
**Main features of the computational models presented.** ^1^Software tools that were used to build the model or previous model that a particular work was based on. The term In-House is used when, to our best effort, we could not find information regarding the implementation of a particular model. We assume it had to be a unique tool that was developed strictly for the scope of the referenced study. ^2^Other tissues or organs where the simulation takes place. The umbrella term Cell is used for intracellular dynamics that, in principle, should apply to any kind of tissue. ^3^Crosstalk and tropisms, but also additional signals or pathways that are modeled as a result of, or coupled to, Auxin expression. ^4^This property describes the nature of the mesh used to represent the tissue involved in the simulation. The letter S represents a static mesh established since the beginning of the model and that remains fixed throughout the whole simulation. A typical example is the lattice of square cells that is used to depict a 2D slice of the root. The letter D is used for a dynamic mesh that can deform and evolve as the simulation goes on. It can be used to represent cells which continuously change in size and geometry. ^5^The spatial context refers to the topology of the space where the simulation takes place. Analytical solutions are considered to be dimensionless. 1.5D refers to a 1D system describing a feature that exists normally in a 2D space, for instance, the edge of a growing leaf. Similarly, a 2.5D model is essentially a 2D model projected onto a 3D space, like the surface of the developing shoot meristem. Additional references for the software tools presented: AuxSim: Kramer, 2004; Berkeley Madonna: http://berkeleymadonna.com; COPASI: Hoops et al., 2006; L-Studio: http://algorithmicbotany.org/virtual_laboratory/; SOFA: Faure et al., 2012; VirtualLeaf: Merks et al., 2011; VV Environment: Smith et al., 2004.

All computational models considered in this perspective are shown in **Figure 1**, along with their properties. The vast majority of them implement auxin diffusion and transport as the core mechanism that drives the computational simulation. Only a few computational models, to date, have considered the effects of a dynamically growing tissue together with its structural properties (e.g., Hamant et al., 2008; Barrio et al., 2013; Sassi et al., 2014).

The corpus of algorithms that the field of computational modeling of auxin has produced is enough to justify the creation of an integral model of plant development. This unified model would account, at least, for all the phenomena reviewed previously. In summary, this is how, we perceive such a model coming to existence. A single cellular layer integrates signals from physical phenomena (i.e., mechanical force, environmental stimuli) and regulatory pathways to ultimately define the metabolism of auxin and its transport. The cellular layer is then used to predict the resulting pattern of auxin present in a multi-cellular tissue which, in turn, signals the reprogramming of cells and the appearance of new organs as well as the vasculature needed to sustain them.

Studies that establish the physical constraints associated with auxin would further improve the validity of this model (Kramer et al., 2007, 2011; Beauzamy et al., 2015; Kramer and Ackelsberg, 2015; Boot et al., 2016). And, many existing software tools that deal with the acquisition of data (Schmidt et al., 2014; Barbier de Reuille et al., 2015) and its visualization (Band et al., 2014) would prove to be useful as well.

Due to its complexity, a model of this nature would have to come from a large collaborative effort among the interested scientific community. We identify two issues that could prevent this from happening, the lack of interoperation between the existing models and the difficulty of extending them with new features. This leads to most efforts producing solitary software packages that are rarely used beyond the scope of their particular study (**Figure 1**). These issues could be mitigated by the establishment of standard modeling conventions and tools, and we anticipate the need for a scientific consortium to coordinate the work of the parties concerned.

With a unified model of plant development, the scientific community would be able to evaluate *in silico* the phenotypical outcome of a given set of initial constraints. Furthermore, it would pave the way for the rational design of new biological structures and functions, a powerful paradigm that, we introduce here as plant engineering.

Plant engineering is a discipline concerned with the application of our current body of knowledge regarding plant development to the design, improvement, and creation of plants to satisfy particular sets of requirements. While this is still a vision set in the far future, we firmly believe that the first step toward it is to establish a common and exhaustive model of plant development built over the foundation laid out by computational modeling of auxin.

## Author Contributions

AM-T and AC-R conceived the layout and scope of the manuscript. AM-T wrote the manuscript with guidance and further editing by AC-R.

## Conflict of Interest Statement

The authors declare that the research was conducted in the absence of any commercial or financial relationships that could be construed as a potential conflict of interest.
